# Effects of Hyperbaric Oxygen Therapy on Long COVID: A Systematic Review

**DOI:** 10.3390/life14040438

**Published:** 2024-03-26

**Authors:** Bing-Qi Wu, De-Yi Liu, Te-Chun Shen, Yu-Ru Lai, Tsai-Ling Yu, Hsiang-Li Hsu, Hsiu-Ming Lee, Wei-Chih Liao, Te-Chun Hsia

**Affiliations:** 1 Department of Education, China Medical University Hospital, Taichung 404, Taiwandeyil845@gmail.com (D.-Y.L.); 038122@tool.caaumed.org.tw (H.-L.H.);; 2 School of Medicine, China Medical University, Taichung 404, Taiwan; u109001307@cmu.edu.tw (Y.-R.L.);; 3 Division of Pulmonary and Critical Care Medicine, Department of Internal Medicine, China Medical University Hospital, Taichung 404, Taiwan; 4 Hyperbaric Oxygen Therapy Center, China Medical University Hospital, Taichung 404, Taiwan; 5 Division of Critical Care Medicine, Chu Shang Show Chwan Hospital, Nantou 557, Taiwan

**Keywords:** coronavirus disease (COVID-19), severe acute respiratory syndrome coronavirus 2 (SARS-CoV-2), long COVID, post-COVID conditions, post-COVID syndrome, hyperbaric oxygen (HBO)

## Abstract

The coronavirus disease (COVID-19) pandemic has resulted in an increasing population that is experiencing a wide range of long-lasting symptoms after recovery from the acute infection. Long COVID refers to this specific condition and is associated with diverse symptoms, such as fatigue, myalgias, dyspnea, headache, cognitive impairment, neurodegenerative symptoms, anxiety, depression, and a sense of despair. The potential of hyperbaric oxygen therapy (HBOT) to improve chronic fatigue, cognitive impairments, and neurological disorders has been established; therefore, the use of HBOT to treat long COVID has also been studied. We conducted a literature search between 1 January 2019 and 30 October 2023, focusing on the clinical efficacy and utility of HBOT for treating long COVID and found ten clinical studies that fit the review topic, including one case report, five one-group pretest-posttest design studies, one safety report from a randomized controlled trial (RCT), and three complete reports of RCTs. Most studies found that HBOT can improve quality of life, fatigue, cognition, neuropsychiatric symptoms, and cardiopulmonary function. Although HBOT has shown some benefits for long COVID symptoms, further rigorous large-scale RCTs are required to establish precise indications, protocols, and post-treatment evaluations.

## 1. Background

### 1.1. What Is Long Coronavirus Disease (COVID)?

The coronavirus disease (COVID-19) pandemic has resulted in a growing population experiencing several long-lasting symptoms following recovery from acute infection. Several medical terms, including “long COVID”, “postCOVID conditions”, and “postCOVID syndrome”, refer to the specific condition. Long COVID is estimated to occur in 20–50% of people with COVID-19 [1]. Long COVID is associated with diverse symptoms, including fatigue, myalgia, dyspnea, headache, cognitive impairment, neurodegenerative symptoms, anxiety, depression, and a sense of despair. The most common symptom is fatigue, presenting in up to 60% of patients with long COVID [1]. “Brain fog” is a set of symptoms, including cognitive impairment, an inability to concentrate and multitask, and short-term and long-term memory loss [2], which have attracted public attention. The pathogenesis of long COVID is largely unknown. However, viral persistence, hypercoagulopathy, immune dysregulation, autoimmunity, hyperinflammation, or a combination of these have been suggested [3]. Currently, no standard treatment has been established for long COVID, and there is a growing requirement for the identification of more effective treatments for this disorder.

### 1.2. Hyperbaric Oxygenation Therapy as a Potential Treatment for Long COVID

Long COVID treatment lacks a high evidence level. Hyperbaric oxygenation therapy (HBOT) is a medical procedure that administers 100% oxygen in a pressurized chamber. As the environmental pressure increases, more oxygen is dissolved into the blood plasma, augmenting the oxygen supply to the tissues. The current indications of HBOT can be broadly classified into three categories: the acceleration of wound healing and enhancement of angiogenesis, enhancement of antimicrobial effects, and some medical emergencies, such as arterial gas embolism and decompression sickness [4]. Emerging research has shown the potential benefits of HBOT in neurological disorders and other syndromes associated with chronic fatigue [5,6]. Several studies have observed improved cognitive functions attributed to HBOT [7,8,9]. Considering our existing knowledge about the symptoms of long COVID, HBOT has emerged as a potential treatment option.

### 1.3. Proposed Mechanism of HBOT on Long COVID

The overall hypothesis of the effect of HBOT on long COVID is that HBOT can reduce oxidative stress and chronic inflammation, improve endothelial dysfunction, and thereby alleviate symptoms associated with long COVID. HBOT can enhance the amount of oxygen dissolved in body tissues; the combined action of hyperoxia and hyperbaric pressure can trigger oxygen- and pressure-sensitive genes, resulting in the induction of regenerative processes, including stem-cell proliferation and mobilization with antiapoptotic and anti-inflammatory factors, angiogenesis, and neurogenesis [10,11,12]. HBOT can improve cerebral blood flow to the malperfused brain regions and microstructure integrity [13,14]; therefore, it can induce neuroplasticity and improve cognitive function. Moreover, HBOT has beneficial effects on mitochondrial function, a crucial element of appropriate muscle function [12]. HBOT can also increase the number of proliferating and differentiating satellite cells as well as regenerating muscle fibers and promoting muscle strength [15,16].

## 2. Materials and Methods

### 2.1. Search Strategy

Shen and Liu independently designed and performed the search strategy using the PubMed, Embase, Cochrane CENTRAL, Web of Science, and ClinicalTrials.gov databases. The search was performed from 1 January 2019 to 30 October 2023. Table 1 shows the detailed search strategy used in this review.

### 2.2. Inclusion and Exclusion Criteria

The inclusion and exclusion criteria were established based on the population, intervention, comparison, and outcome (PICO) principle. The PICO can be described as follows:

- **Population**: People infected with severe acute respiratory syndrome coronavirus 2 (SARS-CoV-2) with long-lasting symptoms (weeks), such as fatigue, myalgia, dyspnea, headache, cognitive impairment, neurodegenerative symptoms, anxiety, depression, and a sense of despair.
- **Intervention**: HBOT.
- **Comparison**: Placebo or not applicable.
- **Outcome**: An assessment of improvements in long COVID symptoms, including fatigue, myalgia, dyspnea, headache, cognitive impairment, neurodegenerative symptoms, anxiety, depression, and a sense of despair, via questionnaires, tests, and scales. Furthermore, any observed enhancements in neuroimages or biochemical markers were considered. All studies were included, irrespective of the definition of long COVID.

This review included randomized controlled trials (RCTs), observational studies, or case reports enrolling human participants with long COVID, characterized by fatigue, myalgia, dyspnea, headache, cognitive impairment, neurodegenerative symptoms, anxiety, depression, and a sense of despair, which were treated with HBOT.

This review was limited to human studies and research papers written in English. Meeting abstracts, reviews, editorials, and opinion articles without data were excluded. Moreover, studies that enrolled participants and overlapped with a previously published trial were excluded to avoid duplicative data. There were no restrictions on age, gender, or ethnicity.

### 2.3. Screening Process and Data Extraction

Two separate authors (Shen and Liu) independently reviewed the titles and abstracts of potential publications acquired through the database search, eliminating any duplicates. The complete text of qualifying articles was subsequently retrieved and subjected to analysis. Each study provided the following data points: authors, country, design, patient count, patient characteristics, HBOT regimen in the treatment group, sham treatment in the control group, evaluation methods, and outcomes. Any discrepancies between authors during the screening and data extraction stages were addressed by a third author for resolution.

### 2.4. Outcomes Evaluation

The participants in these enrolled studies used several evaluation tools, including magnetic resonance imaging-diffusion tensor imaging (MRI-DTI), NeuroTrax computerized tests, cardiopulmonary exercise test, Chalder fatigue scale, ImPACT symptom questionnaire, modified verbal rating muscle and joint pain scale, modified Borg dyspnea scale, venous blood gas test, pulse oximeter, pulmonary function tests, psychotechnical tests, EuroQol instrument, disease-specific symptom questionnaire, numeric rating scale, reactive oxygen species levels, total antioxidant capacity, cytokine concentrations, lipid peroxidation level, DNA damage level, nitric oxide (NO) metabolites, neopterin, creatinine, uric acid, Short-Form-36 questionnaire, visual analog scale, self-reported questionnaires, sense of smell, structural and functional MRI, global longitudinal strain, and myocardial work index parameters.

### 2.5. Methodological Quality Appraisal

To assess the methodological quality of the included studies, the Cochrane risk of bias tool for randomized trials (version 2, RoB 2, London, UK) was employed. This tool comprises six primary items for appraising the study quality, including the randomization process, intervention adherence, missing outcome data, outcome measurement, selective reporting, and overall risk of bias.

Within the intervention adherence segment of RoB 2, two options are presented for literature assessment: intention-to-treat (evaluating intervention assignment) and per-protocol (assessing intervention adherence). For this review, the per-protocol evaluation method was selected because it aligns most suitably with the design of the studies included in our analysis.

## 3. Results

### 3.1. Study Identification and Selection

**Figure 1:**
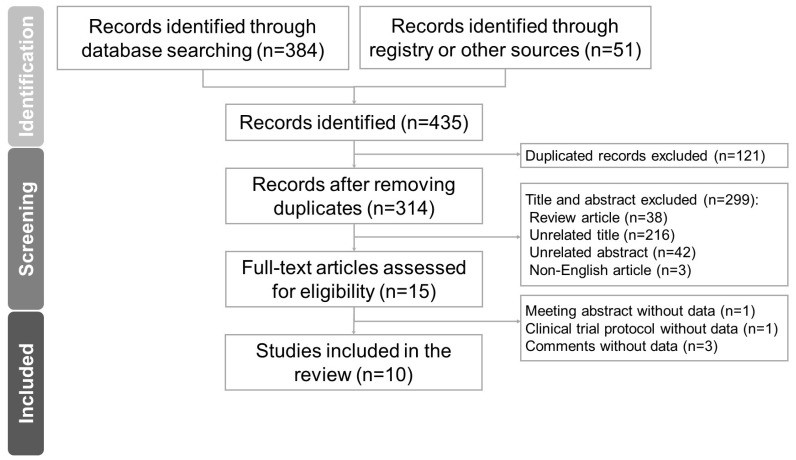
PRISMA flowchart for the current systematic review.

Figure 1 shows the Preferred Reporting Items for Systematic reviews and Meta-Analyses (PRISMA) flowchart of the literature search used in this study. Initially, the electronic search identified 435 potential titles for screening. Following the removal of duplicate articles and exclusion of unrelated articles, based on their titles and abstracts, and articles that did not meet the selection criteria, 10 articles were ultimately included in the study. Of the ten selected articles, one was a case report [17], five were one-group pretest–post-test designed studies [18,19,20,21,22], one was a safety report for RCTs [23], and three were complete reports of RCTs [24,25,26].

### 3.2. Methodological Quality of the Included Studies

**Figure 2:**
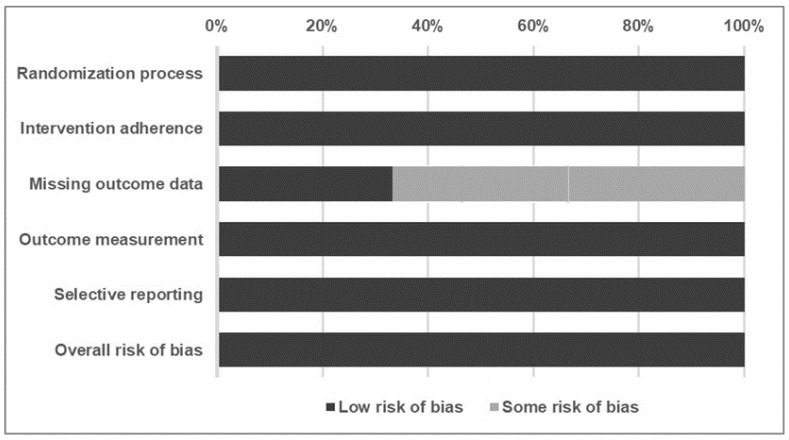
Summary of the quality assessment of studies included in the review using the Cochrane risk of bias 2 tools.

Regarding the overall methodological quality of the included RCTs, two of the evaluated studies [25,26] had some risk of bias and one [24] had a low risk of bias (Figure 2). Table 2 summarizes the risk of bias assessment.

### 3.3. HBOT Application in Long COVID among Non-RCT Trials

Table 3 summarizes the settings and main findings of the current studies of HBOT administration in patients with long COVID. Bhaiyat et al. [17] reported a case of long COVID, characterized by a decline in memory, multitasking abilities, energy, breathing, and physical fitness for 3 months post infection, which was effectively managed with HBOT. The treatment regimen included 60 sessions. Following 15 sessions, the patient’s complaints of fatigue and low energy were reduced. Following 20 sessions, the breathing and exercise capacity and memory and multitasking capabilities had returned to pre-COVID-19 levels. In addition to the patient’s self-reported improvements, brain MRI–DTI conducted 4 weeks following the completion of all HBOT sessions revealed substantial enhancements in brain perfusion and microstructure. The authors proposed that the beneficial effects of HBOT on brain function could be associated with potential neuroplasticity.

In 2021, Robbins et al. [18] conducted a one-group pretest–post-test study with 10 HBOT sessions on 10 patients with newly developed fatigue continuing for >12 weeks during or after COVID-19 infection. The results indicated marked improvements in Chalder fatigue scale scores, global cognition, executive function, attention, information processing speed, and verbal function. In 2022, Zant et al. [19] included six patients with symptoms persisting over 30 days and who were treated with 24–85 HBOT sessions. They found improvements in overall symptoms, joint and muscle pain, and modified Borg dyspnea scale scores. In 2022, Kitala et al. [20] recruited 31 patients, 90% of whom were overweight or obese, and administered them with a cycle of 15 compression sessions. The results revealed a significant improvement in the feelings of anxiety, depression, and perceived pain. Using the Fullerton test, a positive effect on strength, fitness, and flexibility was reported. Moreover, significant changes in the anion gap, lactate levels, and postexercise saturation were reported. Furthermore, a statistically significant improvement in working memory and concentration was observed. Follow-up evaluation data demonstrated that improvements persisted even after the completion of HBOT. Regarding the parameters, the mean quality of life score was observed to be higher than the baseline score. Moreover, subjective experiences of improved physical performance, overall well-being, and energy levels, muscle or joint pain resolution, and improved sleep quality were reported. In 2023, Mrakic-Sposta et al. [21] conducted a one-group pretest–post-test study with 15–50 HBOT sessions on five patients with fatigue, dyspnea, dry cough, and fever for 1–6 months following COVID infection. They evaluated the following numeric rating scales: reactive oxygen species (ROS) levels, total antioxidant capacity, cytokine concentrations from saliva samples, lipid peroxidation, DNA damage, NO metabolites, neopterin, creatinine, and uric acid concentrations from urine samples. The results revealed attenuated ROS production, lipid peroxidation, DNA damage, NO metabolites, and inflammation biomarker levels after HBOT. In 2023, Lindenmann et al. [22] recruited 59 patients with symptoms persisting over 3 months and treated them with 10 HBOT sessions. They found significant improvements in physical functioning, physical role, energy, emotional well-being, social functioning, and pain and a reduced limitation of activities. They indicated HBOT as a promising supportive therapeutic method for long COVID.

In 2022, Kjellberg et al. [27] reported a protocol for a phase II, randomized, placebo-controlled, double-blind clinical trial. This study aimed to explore the potential of HBOT in enhancing the health-related quality of life (HRQoL) in long COVID patients. In 2023, the interim safety report of the trial was announced [23]. The authors noticed that 20 patients received safety evaluations at 13 weeks; 31 adverse events were documented, impacting 60% of study participants. However, no serious adverse events occurred. Cough and chest discomfort were the most common adverse events.

### 3.4. HBOT Application in Long COVID among RCT Trials

Table 4 presents HBOT application in long COVID among RCT trials in details. Zilberman–Itskovich et al. [24] conducted and reported the results of the first prospective, randomized, double-blind, sham-controlled, phase II exploratory study. They described significant group-by-time interactions in global cognitive function, attention, and executive function. These findings were consistent with significant improvements in brain MRI–DTI perfusion and microstructural changes. These outcomes indicate that HBOT can induce neuroplasticity and improve cognitive symptoms in patients with long COVID-19.

Catalogna et al. [25] performed a subgroup analysis based on the same population [24]. In structural and functional MRI–DTI imaging, observable longitudinal alterations in cerebral network connectivity were observed, accompanied by cognitive and emotional recuperation. Furthermore, HBOT-associated cognitive enhancement aligns with the reorganization and recuperation of connectivity modalities within expansive cognitive control networks. After examining the association between cognitive and psychiatric scores, enhanced functional connectivity and structural efficacy parameters of the amygdala circuit may reflect improved psychiatric symptoms. The authors concluded that HBOT contributes to the restoration of white matter tracts and alters the functional connectivity organization of neural pathways correlated with cognitive and emotional recovery in patients with long COVID. They indicated the use of structural and functional connectivity analysis as a selection tool for monitoring HBOT eligibility and response.

Leitman et al. [26] conducted another subgroup analysis from the first RCT [24]. In this study, all patients showed normal conventional echocardiography parameters, except for global longitudinal strain (GLS). A post hoc analysis demonstrated a significant increase in GLS for both the HBOT and sham groups. However, only the HBOT group exhibited a significant increase in global work efficacy, whereas the sham group showed no such increase. Approximately 62.5% of patients achieved normal GLS levels in the HBOT group compared with 38.4% in the sham group. The authors emphasized that HBOT promotes the recovery of left ventricular systolic function in patients suffering from long COVID.

## 4. Discussion

HBOT is not currently included in COVID-19 treatment guidelines; however, it shows potential benefits in addressing the sequelae of COVID-19. Existing research indicates significant improvement in clinical conditions across various parameters, including the quality of life, general physical condition, working memory, concentration, and cardiopulmonary function. Moreover, the research demonstrated the efficacy of HBOT in alleviating and improving cognitive impairment among post COVID-19 patients. These improvements are potentially associated with induced neuroplasticity and reduced neuroinflammation. Furthermore, some researchers reported that these clinical improvements correlated with neuropathy alterations.

### 4.1. Pros and Cons of Enrolled Non-RCT Trials

The advantages of conducting one-group pretest–post-test design studies, framed as pioneering research, are as follows: (1) by using a consistent definition of long COVID, along with uniform HBOT protocols, the study reduces variability and increases the reliability of the results and (2) these studies can provide a solid foundation for future RCTs by demonstrating feasibility and potential efficacy and informing the design of larger studies, including identifying optimal treatment parameters and patient selection criteria. However, several limitations and potential bias may exist in these studies:

1. Lack of a control group: Without a comparison group not receiving HBOT, it is challenging to determine whether improvements are because of the therapy itself or natural recovery over time.
2. Selection Bias: The process of selecting participants from a single geographical area may result in a sample not representative of all long COVID sufferers.
3. Measurement Bias: Using assessment tools to assess symptom improvement might not completely capture the complex and multidimensional nature of long COVID.
4. Placebo Effect: Patients who know they are receiving HBOT might experience subjective improvements because of their expectations of the treatment’s efficacy rather than the treatment itself.
5. Reporting Bias: Participants might report symptom improvements they believe are expected by researchers, especially if the assessment tools involve self-reported measures.
6. Confounding Variables: Other factors, such as concurrent treatments, lifestyle changes, or psychological support, may influence symptom improvement.
7. Small Sample Size: These studies included only some participants; therefore, they may not have sufficient power to detect significant differences before and after HBOT.

In summary, this type of pioneering research plays a critical role in advancing our understanding of HBOT’s potential benefits for patients with long COVID. It sets the stage for more rigorous investigations, informs clinical practice, and eventually contributes to improved patient care.

### 4.2. Pros and Cons of Enrolled RCT Trials

Conducting an RCT on the efficacy of HBOT for long COVID using uniform definitions of long COVID and applying a consistent HBOT protocol presents several pioneering advantages. Using a uniform definition of long COVID permits a focused examination of HBOT effects on a homogenous patient population. By using a consistent HBOT protocol across all participants, these trials set a foundation for reproducibility in future research. Moreover, these trials provide valuable data on the safety and tolerability of HBOT for patients with long COVID. However, several limitations and potential biases may exist in these studies:

1. Selection Bias: Recruiting participants from a single geographic area might limit the generalizability of findings.
2. Blinding Issues: If participants can distinguish between the real and sham HBOT (1.03–1.2 ATA), this could lead to bias in symptom reporting.
3. Measurement Bias: Using assessment tools may not capture the full spectrum of long COVID symptoms or the multidimensional impact of HBOT on patient health.
4. Placebo Effect: Even in a control group, the placebo effect can still influence the outcomes.
5. Reporting Bias: Participants might consciously or unconsciously report improvements that they think the researchers want to hear, especially in subjective assessments of symptoms.
6. Confounding Variables: There could be other factors influencing the outcomes not controlled in the study design, such as concurrent treatments, lifestyle changes, or variations in healthcare access among participants.

Addressing these limitations in future research will be critical for providing more definitive evidence on the efficacy of HBOT for long COVID. These challenges can be overcome by expanding the scope of the study to include diverse populations, extending follow-up periods, and using various objective assessment tools.

### 4.3. Review Limitations

This systematic review thoroughly searched the existing literature on this novel and innovative topic. However, because COVID-19 only emerged a few years ago, there is not yet an abundance of literature from which definitive conclusions can be drawn. Nonetheless, it still provides important information.

The review included ten studies, three of which were RCTs. Beyond considering the limitations of individual studies, several essential issues must be considered for an overall interpretation:

1. Diagnostic criteria and timing for long COVID are inconsistent.
2. The pressure, frequency, and duration of HBOT vary.
3. The assessment tools used were not consistent.
4. The three RCTs involved the same population from the same institution, evaluating different aspects, making it impossible to perform a meta-analysis.
5. Two of the RCTs excluded 23.2% and 17.8% original participants with long COVID. This may affect the accuracy of the results.

## 5. Conclusions

HBOT has shown some benefits for long COVID symptoms. However, the adverse effects of HBOT, such as cough and chest discomfort, may affect patient compliance. Moreover, current evidence is mostly established from case reports or one-group pretest–post-test designed studies. Even from RCTs, the three current RCTs were based on the same patient population. Moreover, the precise timing of the intervention, therapy protocol, and patient heterogeneity may influence outcomes. Therefore, further rigorous, large-scale, RCTs are required to establish indications, protocols, and post-treatment evaluations.

## Figures and Tables

**Table 1:** Keywords and search results in different databases.

Database	Keywords	Filter	Initial Date	Final Date	Results
PubMed	(“hyperbaric” [All Fields] OR “hyperbarics” [All Fields]) AND (“cell respiration” [MeSH Terms] OR “cell” [All Fields] AND “respiration” [All Fields]) OR “cell respiration” [All Fields] OR “oxygenation” [All Fields] OR “oxygen” [MeSH Terms] OR “oxygen” [All Fields] OR “oxygen s” [All Fields] OR “oxygenate” [All Fields] OR “oxygenated” [All Fields] OR “oxygenates” [All Fields] OR “oxygenating” [All Fields] OR “oxygenations” [All Fields] OR “oxygenative” [All Fields] OR “oxygenator s” [All Fields] OR “oxygenators” [MeSH Terms] OR “oxygenators” [All Fields] OR “oxygenator” [All Fields] OR “oxygene” [All Fields] OR “oxygenic” [All Fields] OR “oxygenous” [All Fields] OR “oxygens” [All Fields]) AND (“SARS-CoV-2“ [MeSH Terms] OR “SARS-CoV-2” [All Fields] OR “COVID” [All Fields] OR “COVID-19” [MeSH Terms] OR “COVID-19” [All Fields] OR (“SARS-CoV-2” [MeSH Terms] OR “SARS-CoV-2” [All Fields] OR “SARS-CoV-2” [All Fields]))	NA	1 January 2019	30 October 2023	106
Embase	(‘hyperbaric oxygen’/exp OR ‘hyperbaric oxygen’) AND ((‘coronavirus disease 2019’/exp OR ‘coronavirus disease 2019’) OR (‘SARS-CoV-2’/exp OR ‘SARS-CoV-2’))	NA	1 January 2019	30 October 2023	202
Cochrane CENTRAL	#1 hyperbaric oxygen #2 MeSH descriptor: [hyperbaric oxygen] explode all trees #3 COVID #4 MeSH descriptor: [COVID-19] explode all trees #5 SARS-CoV-2 #6 MeSH descriptor: [SARS-CoV-2] explode all trees #7 (#1 OR #2) AND (#3 OR #4 OR #5 OR #6)	NA	1 January 2019	30 October 2023	41
Web of Science	(ALL = (COVID) OR ALL = (SARS-CoV-2)) AND ALL = (hyperbaric oxygen)	NA	1 January 2019	30 October 2023	76
ClinicalTrials.gov	Condition/disease: COVID Intervention/Treatment: hyperbaric oxygen	Condition or disease, intervention or treatment	1 January 2019	30 October 2023	10

**Table 2:** Detailed quality assessment of the included studies using the Cochrane risk of bias 2 tools.

First Author	Year	Randomization Process	Intervention Adherence	Missing Outcome Data	Outcome Measurement	Selective Reporting	Overall RoB
Zilberman-Itskovich [24]	2022	L	L	L	L	L	L
Catalogna [25]	2022	L	L	S ^1^	L	L	S
Leitman [26]	2023	L	L	S ^2^	L	L	L

^1^ The study excluded nine patients from the HBOT group and eight patients from the control group due to excessive motion or head motion artifacts of MRI which resulted in a potential for underpowered outcome estimation. ^2^ The study excluded seven patients from the HBOT group and six patients from the control group due to unsaved results or inadequate quality of echocardiography which resulted in a potential for underpowered outcome estimation. L: low risk of bias; RoB: risk of bias; S: some concerns.

**Table 3:** A summary of research on HBOT in long COVID.

Author, Country, Year	Study Design	Patient Number	Patient Status	HBOT Regimen in the Treatment Group and Sham in the Control Group	Evaluation	Outcomes and Conclusion
Bhaiyat et al., United Arab Emirates and Israel, 2022 [17]	Case report, with pretest and post-test	1, 55 y/o, male	55-year-old Caucasian man presented with long COVID symptoms, including a decline in memory, multitasking abilities, energy, breathing, and physical fitness for three months after infection	60 sessions (12 weeks), 5 days per week Each session: 90 min of 100% O_2_ at 2 ATA, with 5-min air breaks every 20 min	MRI-DTI; NeuroTrax computerized tests; CPET	**Outcomes** Improvements in brain perfusion and microstructure. Improvement in global memory, with the most dominant effect on nonverbal memory, executive functions, attention, information processing speed, cognitive flexibility, and multitasking. 34% increase in VO2 max; 44% increase in FVC; 23% increase in FEV; and 20% increase in PEF. **Conclusion** HBOT could result in improvements in cognition and cardiopulmonary function.
Robbins et al., UK, 2021 [18]	One-group pretest-posttest design	10, 47.5 y/o, 4 males	Newly developed fatigue continuing for more than 12 weeks during or after infection	10 sessions (2 weeks), 5 days per week Each session: 105 min of 100% O_2_ at 2.4 ATA, with 5-min air breaks every 30 min	Chalder fatigue scale; NeuroTrax computerized tests	**Outcomes** Significant improvement in Chalder fatigue scale (*p* = 0.0059). Significant improvements in global cognition (*p* = 0.0137), executive function (*p* = 0.0039), attention (*p* = 0.0020), information processing speed (*p* = 0.0059), and verbal function (*p* = 0.0098). **Conclusion** HBOT has a positive effect on long COVID-related fatigue and brain fog.
Zant et al., USA 2022 [19]	One-group pretest-posttest design	6, unknown age and sex	Lasting long COVID symptoms for more than 30 days	24–85 sessions (3–12 weeks), 3–5 days per week Each session: 90 min, 100% O_2_ at 2 ATA	ImPACT symptoms questionnaire; modified verbal rating muscle and joint pain scale; modified Borg dyspnea scale	**Outcomes** Improvements in overall symptoms; significant improvements in numbness or tingling (*p* < 0.001), trouble falling asleep (*p* < 0.001), feeling slowed down (*p* < 0.001), fatigue (*p* = 0.006), drowsiness (*p* < 0.001), feeling more emotional (*p* = 0.004), nervousness (*p* = 0.004), irritability (*p* = 0.03), difficulty remembering (*p* = 0.03), sleeping less than usual (*p* = 0.014), feeling mentally foggy (*p* = 0.009), difficulty concentrating (*p* = 0.022), and balance problems (*p* = 0.034). Improvement in joint and muscle pain. Significant improvements in modified Borg dyspnea scale (*p* < 0.001). **Conclusion** HBOT could improve lingering COVID-19 respiratory and neurological symptoms.
Kitala et al., Poland, 2023 [20]	One-group pretest-posttest design	31, 55 y/o, unknown sex	Fatigue, weakness; bone, muscle, and joint pain; breath, concentration, memory, and sleep problems; headache and nervousness; for more than three weeks after the resolution of COVID-19	15 sessions (3 weeks), 5 days per week Each session: 75 min of 100% O_2_ at 2.5 ATA	Venous blood gas test; pulse oximeter before and after Fullerton test; spirometry tests; psychotechnical tests; EQ-5D; disease-specific symptom questionnaire	**Outcomes** Significant improvement in quality of life (*p* < 0.001), walking problems (*p* = 0.00033), self-service problems (*p* = 0.01417), daily activities (*p* = 0.00058), sense of anxiety and depression (*p* = 0.00011), and pain (*p* = 0.00274). Significant improvements in other symptoms, including headache (*p* < 0.00001), fatigue (*p* < 0.00001), muscle pain (*p* < 0.00001), joint pain (*p* < 0.00001), sleep problems (*p* < 0.00001), and blood pressure spikes and heart rate fluctuations (*p* = 0.03284). Significant improvement in cognitive functions, including working memory (*p* = 0.01547), concentration (*p* < 0.0001), and attention (*p* = 0.03297). Significant decrease in lactate (*p* = 0.02628) and anion gap (*p* = 0.01036). Significant increase in oxygen saturation after Fullerton test (*p* = 0.03208). Significant changes in FVC (*p* = 0.03182). Significant improvements in two-minute step test (*p* < 0.00001), standing up from a chair in 30 s test (*p* < 0.00001), and sit-up and reach test (*p* = 0.00023). **Conclusion** HBOT results in improvements in quality of life, physical resilience and power, targeted gasometric and spirometric parameters, and working memory and attention.
Mrakic-Sposta et al., Italy, 2023 [21]	One-group pretest-posttest design	5, 41.2 years old, 3 males	28–55 year-old patients Fatigue, dyspnea, dry cough, fever for 1–6 months after infection	2 for 15 sessions (3 weeks), 5 days per week Each session: 90 min of 100% O_2_ at 2.4 ATA 2 for 30 sessions (6 weeks), 5 days per week Each session: 90 min of 100% O_2_ at 2.4 ATA 1 for 50 sessions (10 weeks), 5 days per week Each session: 90 min of 100% O_2_ at 2.4 ATA	Numeric rating scale, reactive oxygen species levels, total antioxidant capacity, cytokine concentrations (IL-6, IL-1, and TNF-α), lipid peroxidation (8-isopGF2α), DNA damage (8-OH-dG), nitric oxide metabolites, neopterin, creatinine, and uric acid	**Outcomes** Attenuated ROS production, lipid peroxidation, DNA damage, NO metabolites, and inflammation biomarker levels after HBOT. **Conclusion** HBOT may represent an alternative non-invasive method for treating long COVID.
Lindenmann et al., Austria, 2023 [22]	One-group pretest-posttest design	59, 43.9 years old, 26 males	18–90 year-old patients A minimum of two typical symptoms for more than 3 months after infection	10 sessions (2 weeks), 5 days per week Each session: 75 min of 100% O_2_ at 2.2 ATA	Short-Form-36 questionnaire (SF-36); visual analog scale (VAS)	**Outcomes** Significant improvements in physical functioning (*p* < 0.001), physical role (*p* = 0.01), energy (*p* < 0.001), emotional well-being (*p* < 0.001), social functioning (*p* < 0.001), pain (*p* = 0.01), and reduced limitation of activities (*p* < 0.001). **Conclusion** HBOT as a promising supportive therapeutic tool for the treatment of long COVID.
Kjellberg et al., Sweden, 2023 [23]	Interim safety report from a randomized, sham-controlled, double-blind trial	20, unknown age, 2 males	18–60 year-old, previously healthy patients Symptoms consistent with long COVID for a minimum of 12 weeks Diagnosis with ICD-10 code U09.9 (long COVID)	**Treatment group (n = 8)** Maximum 10 sessions within 6 weeks Each session: 90 min, 100% O_2_ at 2.4 ATA, with two 5-min air breaks **Control group (n = 12)** Maximum 10 sessions within 6 weeks Each session: 90 min, air at 1.35 ATA, with two 5-min air breaks	Primary evaluation: physical domains, physical functioning and physical role in RAND-36. Second evaluation: objective physical tests including 6-min walk test and 30-s chair stand, EQ-5D, and reactive hyperemia index. Safety evaluation: occurrence, frequency, and seriousness of adverse events.	**Outcomes** Primary and secondary endpoint data were not reported. 20 subjects had safety evaluation data (at 13 weeks). In total, 31 adverse events were documented, impacting 60% of study participants. No serious adverse event was reported. All adverse events were temporary and predominantly mild, with six of them being moderate. Cough and chest discomfort were the most common. **Conclusion** HBOT appears to have a favorable safety profile for post-COVID conditions.
Zilberman-Itskovich et al., Israel, 2022 [24] * (1 February 2022 completed)	Randomized, sham-controlled, double-blind trial	73, 48.1 years old, 29 males	Over 18 years old Cognitive symptoms for more than three months after symptomatic COVID-19	**Treatment group (n = 37):** 40 sessions (2 months), 5 days per week Each session: 90 min, 100% O_2_ at 2 ATA, with 5-min air breaks every 20 min Compression and decompression rates: 1.0 m/min **Control group (n = 36):** 40 sessions (2 months), 5 days per week Each session: 90 min, air at 1.03 ATA (chamber pressure was raised to 1.2 ATA during the first five minutes of the session along with circulating air noise followed by decompression (0.4 m/min) to 1.03 ATA during the next five minutes)	Primary evaluation: NeuroTrax computerized tests. Secondary evaluation: MRI-DTI, self-reported questionnaires, sense of smell, pulmonary function.	**Outcomes** Significant group-by-time interaction in global cognitive score (*p* = 0.038), attention (*p* = 0.04), and executive function (*p* = 0.05). Significant improvements in physical limitations (*p* = 0.023), energy (*p* = 0.029), sleep (*p* = 0.042), psychiatric symptoms (*p* = 0.008), somatization (*p* = 0.014), depression (*p* = 0.04), and pain interference (*p* = 0.001). Significant improvements in brain perfusion (*p* < 0.0005) and microstructures (*p* < 0.002). **Conclusion** HBOT improves dysexecutive functions, psychiatric symptoms, pain interference symptoms, and fatigue in patients suffering from long COVID.
Catalogna et al., Israel, 2022 [25] * (8 August 2022 completed)	Randomized, sham-control, double-blind trial	56, 48.2 years old, 22 males	Over 18 years old Cognitive symptoms for more than three months after a symptomatic COVID-19 **Without excessive motion and head motion artifacts**	**Treatment group (n = 28)** **Control group (n = 28)** The same as above study	Structural and functional MRI imaging	**Outcomes** Decreased internetwork connectivity in the HBOT group, which was negatively correlated to improvements in attention and executive function scores (*p* < 0.001). Significant group-by-time interactions in the right hippocampal resting state functional connectivity in the medial prefrontal cortex (*p* = 0.002). Negative correlation in the brief symptom inventory score and in the resting state function connectivity between the amygdala seed, the angular gyrus, and the primary sensory motor area (*p* = 0.012 and 0.002). Positive correlations between the brief symptom inventory score and the left insular cortex seed and angular gyrus (*p* < 0.0001). Significant group-by-time interaction in the fractional anisotropy of left amygdala tracts (*p* = 0.007) and in the amygdala circuit (*p* = 0.017). **Conclusion** HBOT improves disruptions in white matter tracts and alters functional connectivity organization of neural pathways, attributed to the cognitive and emotional recovery in patients suffering from long COVID.
Leitman et al., Israel, 2023 [26] * (8 August 2022 completed)	Randomized, sham-control, double-blind trial	29, unknown age and sex	Over 18 years old Cognitive symptoms for more than three months after symptomatic COVID-19 **With adequate echocardiography and reduced GLS at baseline**	**Treatment group (n = 16):** **Control group (n = 13)** The same as above study	Primary evaluation: GLS. Secondary evaluation: myocardial work index parameters including GWI, GCW, GWW, and GWE.	**Outcomes** Increase GLS in the HBOT group and sham group, with significant group-by-time interaction (*p* = 0.041). Significant increase in GWE in the HBOT group (*p* = 0.02) but not in the sham group. A total of 62.5% of the patients achieved normal GLS levels in the HBOT group compared to 38.4% in the sham group (*p* = 0.08). **Conclusion** HBOT promotes left ventricular systolic function recovery in patients suffering from long COVID.

* Data reported from the same clinical trial in the same institute. Abbreviation: ATA, atmosphere absolute; CPET, cardiopulmonary exercise test; COVID, coronavirus disease; DTI, diffusion tensor imaging; FEV, forced expiratory volume; FVC, forced vital capacity; GCW, global constructive work; GLS, global longitudinal strain; GWE, global work efficacy; GWI, global work index; GWW, global wasted work; HBOT, hyperbaric oxygen therapy; MRI, magnetic resonance imaging; PEF, peak expiratory flow; VO2, oxygen consumption.

**Table 4:** A detailed summary of RCTs of HBOT in patients with long COVID.

Authors	N	n (Exclusion)	Study Interests	Evaluations	Outcomes
Zilberman-Itskovich et al. [24]	73	**Cases = 37** **Controls = 36**	**Neurocognitive functions** **and symptoms**	Primary: **NeuroTrax computerized tests** Secondary: MRI-DTI, self-reported questionnaires, sense of smell, pulmonary function	**Outcomes** Significant group-by-time interaction in global cognitive score, attention, and executive function. Significant improvements in energy domain, sleep, psychiatric symptoms, and pain interference. Significant improvements in brain perfusion and microstructures. **Conclusion** HBOT improves dysexecutive functions, psychiatric symptoms, pain interference symptoms, and fatigue in patients suffering from long COVID.
Catalogna et al. [25]	73	**Cases = 28** **Controls = 28** (17 patients excluded due to MRI images not meeting criteria)	**Functional and structural connectivity**	**Structural and functional MRI**	**Outcomes** Decreased internetwork connectivity in the HBOT group, which was negatively correlated to improvements in attention and executive function scores. Significant group-by-time interactions in the right hippocampal resting state functional connectivity in the medial prefrontal cortex. Negative correlation in the brief symptom inventory score and in the resting state functional connectivity between the amygdala seed, the angular gyrus, and the primary sensory motor area. Positive correlations between the brief symptom inventory score and the left insular cortex seed and angular gyrus. Significant group-by-time interaction in the fractional anisotropy of left amygdala tracts and in the amygdala circuit. **Conclusion** HBOT improves disruptions in white matter tracts and alters functional connectivity organization of neural pathways, attributed to cognitive and emotional recovery in patients suffering from long COVID.
Leitman et al. [26]	73	**Analysis 1:** **Cases = 30** **Controls = 30** (13 patients excluded due to echocardiography results not meeting criteria)	**Myocardial function**	Primary: **GLS** Secondary: GWI, GCW, GWW, GWE	**Outcomes 1** Increase in GLS in the HBOT group and sham group, without significant group-by-time interaction. No other significant changes between the two groups.
		**Analysis 2:** **Cases = 16** **Controls = 13** (13 patients excluded due to echocardiography results not meeting criteria) (31 patients excluded due to not having reduced GLS at baseline)			**Outcomes 2** Increase in GLS in the HBOT group and sham group, with significant group-by-time interaction. Significant increase in GWE in the HBOT group but not in the sham group. A total of 62.5% of the patients achieved normal GLS levels in the HBOT group compared to 38.4% in the sham group. **Conclusion** HBOT promotes left ventricular systolic function recovery in patients suffering from long COVID.

DTI, diffusion tensor imaging; GCW, global constructive work; GLS, global longitudinal strain; GWE, global work efficacy; GWI, global work index; GWW, global wasted work; MRI, magnetic resonance imaging.

## Data Availability

All data generated or analyzed during this study are included in this manuscript.
